# The gene-modulating power of Tannins isolated from *Jatropha integerrima* flowers on the transcriptomic profile of multidrug-resistant *Klebsiella pneumoniae*

**DOI:** 10.1038/s41598-025-32490-0

**Published:** 2026-01-14

**Authors:** Fatma S. Mahrous, Shimaa M. Khalifa, Fatma Sayed Abdel-Aal Farag, Mona Shaban E. M. Badawy, Omnia Karem M. Riad, Mona H. Ibrahim, Maha M. Soltan, Mohamed Marzouk

**Affiliations:** 1 Department of Pharmacognosy and Medicinal Plants, Faculty of Pharmacy (Girls), Al-Azhar University, Cairo, 11754 Egypt; 2 Department of Microbiology and Immunology, Faculty of Pharmacy (Girls), Al-Azhar University, Cairo, 11754 Egypt; 3 Department of Pharmaceutical Medicinal Chemistry and Drug Design, Faculty of Pharmacy (Girls), Al-Azhar University, Cairo, 11754 Egypt; 4 Biology Unit, Central Laboratory for Pharmaceutical and Drug Industries Research Institute, Chemistry of Medicinal Plants Department, National Research Centre, 33 El Bohouth St. (Former El-Tahrir St.), Dokki, Cairo, 12622 Egypt; 5 Chemistry of Tanning Materials and Leather Technology Department, Chemical Industries Research Institute, National Research Centre, 33 El-Bohouth St. (Former El-Tahrir St.), Dokki, Cairo, 12622 Egypt

**Keywords:** *Jatropha integerrima*, Euphorbiaceae, Ellagitannins, NMR, Gene-modulating power, Multidrug-resistant *Klebsiella pneumonia*, Biofilm, Microbiology, Plant sciences

## Abstract

The continuous increase in antibiotic resistance necessitates a global need to search for new sustainable antimicrobial agents, such as plant-delivered antimicrobial components. This study investigates the antimicrobial and antibiofilm properties of two tannins isolated from the flowers of *Jatropha integerrima* against multidrug-resistant *Klebsiella pneumoniae*. The two active tannins were isolated from the methanol-soluble portion of 70% aqueous methanol flower extract, by consecutive column chromatography. Their antimicrobial activity against the resistant *K. pneumoniae* isolate (BKP-122) was assessed through agar well diffusion and minimum inhibitory concentration (MIC) assays. Their antibiofilm activity was evaluated by using the microtiter plate method and real-time PCR to reveal their effect on the biofilm-associated genes.

Their structures were identified as 2 hydrolysable ellagitannins, namely 1-*O*-galloyl-3,6-(*R*)-hexahydroxydiphenoyl-**D**-B_1,4_-glucopyranose (Jatrophenin-1) and 1-*O*-galloyl-3,6-(*R*)-valoneoyl-**D**-B_1,4_-glucopyranose (Jatrophenin-2), together with vicenin-2, acacetin 7-*O*-*β*-**D**-glucopyranoside, (*E*)-*p*-coumaric acid, and sucrose, based on the chromatographic characters, ^1^H-, and ^13^C NMR analyses. They were found to have potent antimicrobial activity and antibiofilm activity against the resistant *K. pneumoniae* isolate (BKP-122). Real-time PCR analysis indicated that treatment with tannins resulted in the downregulation of critical biofilm-related genes, including *luxS*, *mrkA*, *pgaA*, *wzm*, and *wbbM*. Additionally, the two compounds showed high docking binding scores against Topoisomerase IV, KPLpxH, and *β*-lactamase enzymes, and stable complexes, as evidenced by binding energy values ranging from -8.2 to -10 kcal/mol. These findings underscore the effectiveness of *J. integerrima* tannins as a viable therapeutic strategy to combat antibiotic resistance and biofilm-related infections, highlighting their role in modulating bacterial gene expression and biofilm development.

**Supplementary Information:**

The online version contains supplementary material available at 10.1038/s41598-025-32490-0.

## Introduction

Medicinal plants have been used for centuries in the treatment of various human illnesses. Over time, herbal remedies have been transformed into therapeutic agents that demonstrate significant potential in their effectiveness. Plants exert curative power through their ability to biosynthesize a wide array of bioactive compounds (secondary metabolites)^1^.

*Jatropha integerrima* Jacq., commonly known as spicy Jatropha or peregrina, is a member of the Euphorbiaceae family, a group renowned for its diverse phytochemical constituents and medicinal value. It is native to the Caribbean and Central America, and widely cultivated in tropical and subtropical regions^2,3^. As antimicrobial plant sources, several Euphorbiaceae species have been proven to effectively inhibit various bacterial infections^4^. Moreover, *J. integerrima* is an ornamental shrub that has recently garnered attention in the pharmacological research field as a rich reservoir of bioactive compounds, particularly its flowers^5–7^.

It is worth mentioning that the flowers are not only aesthetically valued but also chemically significant. Phytochemical investigations have revealed a broad range of their secondary metabolites, such as flavonoids, tannins, alkaloids, and phenolic acids^7,8^, that are studied for their antimicrobial, antioxidant, anti-inflammatory, and hepatoprotective properties^9–11^. Specifically, the tannins have emerged as potent agents for the treatment of a wide range of biological effects, including antimicrobial activity against resistant bacterial strains^12,13^ and cytotoxicity^12^. Meanwhile, several biological activities, including the antimicrobial properties, were predicted at bio safe quantities^14^.

Recent studies have begun to explore the potential of plant-derived polyphenols, such as those found in *J. integerrima* flowers, to modulate bacterial behavior at the molecular level^15^. This includes the disruption of quorum-sensing systems^16,17^, inhibition of biofilm formation^18,19^, and alteration of gene expression in pathogenic microorganisms^20^. These properties explored the position of *J. integerrima* flowers as a promising candidate for developing novel strategies to combat antimicrobial resistance as a growing global health concern.

Despite its traditional use in folk medicine for treating wounds, inflammation, and microbial infections, the molecular mechanisms underlying the biological activity of *J. integerrima* flower constituents remain underexplored. In particular, the potential of its tannin-rich extracts to modulate bacterial transcriptomes and inhibit biofilm development in multidrug-resistant pathogens like *Klebsiella pneumoniae* offers an exciting new avenue for research and therapeutic innovation. The current study aims to isolate and characterize tannins from *J. integerrima* flowers and to evaluate their antibacterial and antibiofilm activities against multidrug-resistant *Klebsiella pneumoniae*. It also investigates the gene-modulating effects of these tannins on the transcriptomic profile of key biofilm-associated genes, including luxS, mrkA, pgaA, wzm, and wbbM. Additionally, the study assesses the biosafety of the tannins by determining their cytotoxicity against a normal cell line, specifically the retinal pigmented epithelium (hTERT-RPE1)^21^.

## Materials and methods

### Plant material

Fresh flowers of *J. integerrima* Jacq. collected from El-Orman Garden, Giza Governorate, in June 2023, Egypt (30° 01′ 44.5" N and 31° 12′ 46.7" E). Dr. Tearse Labib, Botany Specialist, Department of Flora and Taxonomy, El-Orman Botanical Garden, Giza, Egypt, established the identification and authentication of the collected plant. A voucher specimen of the plant (Voucher No. 18) was deposited at the Herbarium of the Pharmacognosy and Medicinal Plants Department, Al-Azhar University, Faculty of Pharmacy, Cairo, Egypt. On July 3rd, 2023, the plant name was verified with http://www.theplantlist.org/tpl1.1/record/kew-104678, accessed on April 11, 2025. The plant collection process complied with all relevant regulations and guidelines.

### Extraction and isolation

A quantity of 1.5 kg powder of air-dried flowers extracted with 70% aqueous methanol (8 × 3 L, 1.5 h, 70 °C); the extract was dried via a rotatory evaporator at 60 °C (Buchi Co., Switzerland) to afford 220g (14.67%) total extract. Thereafter, it is defatted using hot petroleum ether under reflux (4 × 1L, 1.5 h, 60 °C), then taken with hot pure methanol (6 × 1L, 1 h, 70 °C) to get rid of sugar and inorganic salts, affording 185g (12.33%) dry methanol–soluble portion (MSP). An amount of 170 g from the MSP was applied to a polyamide 6S column chromatography (CC) using H_2_O, then H_2_O/MeOH step–gradient up to pure MeOH. Using paper chromatography (PC), UV–light (254/365 nm, Desaga Heidelberg-Germany), and spray reagents, similar fractions are collected into 8 ones (I-VIII). Fractions I (20 g) and II (15 g) were found to be polyphenolic-free–free because of their negative response to the FeCl_3_ test and 2D-PC/FeCl_3_. Fraction III (7.8 g) was further successively fractionated on Sephadex LH–20 using EtOH as an eluent to give 10 fractions (20 mL, each). The eluted fractions were checked by Co-PC and collected into two sub-fractions. The first major sub-fraction gives a pure sample of compound **2** (27 mg). Fraction IV (8.2 g) was isolated on a Sephadex LH-20 column using EtOH as an eluent to give 8 sub-fractions. Thereafter, the major significant sub-fraction was purified twice on Sephadex LH–20 using BIW (BuOH-Isopropanol-H_2_O, 4: 1: 5 Upper layer) followed by MeOH to afford compound **1** (2 g). Fraction V (6.5 g) was further separated by successive fractionation on a Sephadex LH-20 column using ethanol as the eluent, yielding 10 individual fractions (each 20 mL). These fractions were analyzed using Co-PC and then combined into four sub-fractions based on their profiles. The major sub-fraction was subsequently purified twice on Sephadex LH-20, first with BIW and then with methanol, resulting in the isolation of compounds **3** (7 mg), **4** (5 mg), **5** (2 mg), and **6** (10 mg).

### Analytical and chromatographic procedures

^1^H and ^13^C NMR analyses were performed on a Bruker High-Performance Digital FT-NMR Spectrometer Advance III (500 and 125 MHz for ^1^H and ^13^C, respectively). CC was performed mainly on polyamide 6S (Reidel De Haen AG, Seeize, Hannover) and Sephadex LH-20 (Pharmacia, Uppsala, Sweden). The separation and purification processes were controlled by PC analyses (2D- and Co-PC were run on Whatman™ paper No. 1 sheets (46 × 57 cm) delivered from GE Healthcare UK Ltd. (Buckinghamshire, UK) using solvent systems BAW (S1: *n*-BuOH/AcOH/H_2_O; 4: 1: 5, v/v/v, upper layer) and aqueous AcOH (S2: 15%). BIW (*n*-BuOH/2-propanol/H_2_O; 4:1:5, v/v/v top layer) was applied for Sephadex CC. All reagents and solvents (analytical grade) were used from Fisher Chemical (Loughborough, UK).

### Cytotoxicity against normal cells

The immortalized retinal pigmented epithelium, hTERT-RPE1, was kindly provided by Prof. Stig Linder, Karolinska Institute, Stockholm, Sweden, and was used as an in vitro model to investigate the cytotoxic effects against normal cells^21^. Two–fold dilution series were prepared separately, from compounds **1** and **2,** to finally examine each of them at a concentration range of 3.91–125 µg/ml.

### Bacterial strains and culture conditions

A previously recorded biofilm-forming *K. pneumoniae* clinical isolate (BKP-122) exhibited resistance to at least one antibiotic from three distinct antibiotic classes (cephalosporin, aminoglycosides, and carbapenems), categorized as multidrug-resistant (MDR) from a previous study^22^.

Bacterial culture was refreshed from glycerol stock at nutrient–rich Luria Bertani (LB) broth supplemented with 1% w/v glucose and incubated for 18 h at 37 °C while shaken (180 rpm). Tryptic soy agar (TSA) plates were streaked with BKP-122, then incubated for 24 h at 37 °C.^22^.

### Agar well diffusion

Compounds were evaluated in vitro against the BKP-122 using the agar well diffusion method. This technique applied 0.5 McFarland (1.5 × 10^8^ CFU/ml) of microbial isolate to the entire surface of Muller-Hinton agar plates. Next, a 100 µL volume of each compound (at 0.75 mg/ml, in dimethyl sulfoxide (DMSO) was added to each well after an 8 mm diameter hole had been aseptically punched with a sterile corkborer. In synchronization, DMSO was replaced with the compound dilutions to serve as a positive control. The Plates were then after, incubated at 37 °C for 24 h. The antimicrobial activity was indicated by measuring the inhibition zone in millimeters in comparison with the standard antimicrobial drugs, Ceftazidime, Gentamicin, and Ertapenem^3,23^.

### Minimum inhibitory concentration (MIC)

The broth micro dilution method was used to determine the MIC extent of the target compounds (**1** and **2**). Using cationically adjusted Mueller–Hinton broth (CAMHB), the bacterial suspension was made and diluted to half the McFarland standard turbidity (1.5 × 10^8^ colony-forming units (CFU)/ml). Compounds were serially diluted two times in 96-well microtiter plates using CAMHB to achieve a final concentration range of 0.125–8 µg/ml. BKP-122 suspension was added to the plates at a final count of 5 × 10^5^ CFU/ml. The wells devoid of the bacterial inoculum were regarded as negative controls. On the other hand, other wells served as positive controls, were kept free of the tested agents, but included DMSO to solubilize the compounds^22^. The MIC was the lowest concentration that inhibited microbial growth. The experiments were repeated three times in duplicate.

### Antibiofilm activity against BKP-122

The microtiter plate method was used to quantitatively detect the promising compounds’ antibiofilm activity. In this process, 100 μl of the microbial suspension in Muller-Hinton broth with a density of 10^8^ CFU \ml was added to each well of a 96-well, flat-bottom polystyrene tissue culture plate (Sigma-Aldrich Co. LLC, USA). This was followed by the addition of 100 μl from the prepared sub-MIC of **1** and **2** (1/2, 1/4, 1/8, and 1/16 MICs) to each corresponding well. In synchronize, DMSO was replaced the compounds dilutions to serve as positive control while all plates were incubated at 37 °C for 24 h. Following incubation, the plate contents were taken out, and the wells were cleaned three times with phosphate buffer saline (250 μl, pH 7.4) before being dried for 1 h at 60 °C. The wells are then stained for 15 min. using crystal violet (200 μl, 0.1% w/v)^24,25^.After being repeatedly cleaned with distilled water, the plates were left to dry. 200 μl of 33% acetic acid was used to resolubilize the wells. A microplate reader (Tecan Elx800, USA) set to 630 nm was used to measure the optical density of each well. The test was run in triplicate, and subsequent computations were based on their averages^26,27^. The following equation was applied to determine the percentages (%) of **1** and **2** that inhibited biofilms^24^:

% inhibition = 1 − (OD sample/OD positive control) × 100.

### Effect on the transcriptome of genes involved in the formation of biofilms in *BKP-122*

Real-time PCR was used to identify the presence and transcriptomic profile of the BKP-122 biofilm formation genes (*luxS, mrkA, pgaA, wzm, and wbbM*) (Table **S1**). Following the manufacturer’s instructions, total RNA was extracted from the BKP-122 using the TRIzol Reagent (15,596,026, Life Technologies, USA). The total RNA was reverse-transcribed into single-stranded complementary DNA using the Quanti-Tect Reverse Transcription Kit (Qiagen, USA). The forward and reverse gene-specific PCR primers of the above-mentioned genes are displayed in Table S1; 23S rRNA is used as the reference gene (internal control). Furthermore, the sample underwent duplicate real-time PCR, and the mean values of the duplicates were utilized for further analysis. Relative gene expression was estimated using the ΔΔCt method, where fold change equaled (2^−ΔΔCT^)^22^.

1 $$\begin{aligned} 2^{{ - \Delta \Delta {\mathrm{CT}}}} = & \left[ {\left( {{\mathrm{CT}}_{{{\text{gene of interest}}}} - {\text{ CT}}_{{{\text{internal control}}}} } \right){\text{sample A}}} \right. \\ & - \left[ {\left( {{\mathrm{CT}}_{{{\text{gene of interest}}}} - {\text{ CT}}_{{{\text{internal control}}}} } \right)\left. {{\text{sample B}}} \right)} \right] \\ \end{aligned}$$

Where, Sample A: treated sample and Sample B: untreated sample (Positive control).

2 $${\text{Fold change due to treatment }} = \, - {1}/{2}^{{ - \Delta \Delta {\mathrm{CT}}}}$$

#### Statistical analysis

All the values are averages of either duplicate or triplicate experiments. Data are presented as a mean ± standard deviation (SD). The significant differences between the obtained MIC means of compounds **1** and **2** were determined by the unpaired Student’s t-test at a 0.05 probability level. Quantitative data from gene expression assays are often normalized by transcription levels of the reference gene (internal control).

#### Molecular docking study

A molecular docking investigation of the compounds **1** and **2** was performed on Topoisomerase IV (pdb: 7LHZ)^28^, KPLpxH (pdb: 8QK2)^29^, and β-lactamase (pdb: 2ZD8)^30^ using AutoDock 4.2^31^. The production of ligand/protein files, grid, and docking parameter files was executed in accordance with previous studies^32^. The crystal structures of the three proteins were obtained from the Protein Data Bank. A 3D grid box of 60x × 60y × 60z Å, with a spacing of 0.40 Å, is centered at 86.27, −54.20, and 60.76 Å for docking with Topoisomerase IV; at 1.31, 12.74, and 7.94 Å for docking with KPLpxH; and at 9.86, 34.78, and 3.34 Å for docking with β-lactamase. DSV was utilized to evaluate and present the docking results^33,34^.

## Results

### Identification and structure elucidation of the isolated compounds

Six compounds were isolated and identified mainly by ^1^H-, and ^13^C NMR analyses, including two rare hydrolysable ellagitannins for the second time in nature, namely Jatrophenin-1 and -2 (**1**, **2**) alongside four known ones. Chromatographic characters and NMR spectral data of **1** and **2** were given below:

#### Jatrophenin-1(**1**)

Off-white amorphous powder (2 g). *R*_f_ = 0.21 (S1), 0.52 (S2) on PC; deep purple spot turned to pink-red, indigo-red, and blue color with KIO_3_, HNO_2_, and FeCl_3_ spray reagents, respectively. UV (MeOH): *λ*_max_ 220, 265, 286sh nm. ^1^H and ^13^C NMR data (500/125 MHz, DMSO-*d*_6_) were presented in Table 1, Fig. S1 and Fig. S2.

**Table 1:** ^1^H and ^13^C NMR data of Jatrophenins 1 and 2 (500/125 MHz, DMSO-d_*6*_)

No	*1*		Marzouk et al., 2012^35^		*2*		Marzouk et al., 2012^35^	
	*δ*_*H*_	*δ*_*C*_	*δ*_*H*_	*δ*_*C*_	*δ*_*H*_	*δ*_*C*_	*δ*_*H*_	*δ*_*C*_
*B *_*1,4*_-*Glucose*								
1	6.21 *d* (7.5)	92.65	6.22 *d* (7.1)	92.13	6.20*d* (7.0)	92.65	6.24 *d* (7.9)	91.80
2	3.88*br d* (7.5)	71.99	3.89 *br d* (7.1)	71.50	3.88*br d* (7.5)	72.00	3.70 *br d* (6.8)	71.77
3	4.59 *br s*	77.91	4.62 *br s*	77.31	4.59*br s*	77.93	4.53 *br s*	79.48
4	4.22*br s*	62.60	4.23 *br s*	62.06	4.22*br s*	62.60	4.14 *br s*	63.04
5	4.36 *t-like* (8.5)	76.76	4.37 *t-like* (8.2)	76.25	4.35 *t-like* (8.5)	76.77	4.35 *t-like* (8.7)	76.11
6a 6b	4.23 *dd* (11.5, 8.0) 3.96*dd* (10.9, 8.9)	64.39	4.24 *dd* (11.2, 7.9) 3.98 *dd* (10.9, 8.9)	63.86	4.22* m* 3.96*dd*(10.5,8.5)	64.39	4.18 m 3.98 *br t-like* (10.2)	64.06
*Galloyl*								
1′		119.13		118.64		119.13		118.99
2′,6′	7.01 s	109.49	7.03 s	108.93	7.01* s*	109.45	7.04 s	108.32
3′,5′		146.01		145.49		146.01		145.78
4′		139.47		138.80		139.47		139.19
7′		165.31		164.72		165.30		165.22
HHDP/Val								
1′′,1′′′		116.25, 115.95		115.71, 115.42		116.25, 120.86		115.77, 117.89
2′′,2′′′		124.32, 123.55		123.82, 123.01		123.55, 124.32		122.94, 123.74
3′′	6.49	107.40	6.51 s	106.88	6.56* s*	107.40	6.55 s	108.40
4′′,4′′′		145.29, 145.20		144.73, 144.64		145.20, 148.78		144.50, 146.00
5′′,5′′′		135.99, 135.84		135.44, 135.31		136.74, 135.99		136.20, 136.10
6′′,6′′′		144.73, 144.40		144.21, 143.89		14,529, 145.82		144.66, 145.77
7′′,7′′′		167.58, 167.22		166.99, 166.64		167.58, 167.96		167.20, 167.44
3′′′	6.56 s	106.45	6.58 s	106.01	6.49 s	106.45	6.49 s	106.21
1′′′′						115.95		114.70
2′′′′						144.74		142.47
3′′′′					6.92* s*	135.84	6.94 s	135.73
4′′′′						138.44		139.20
5′′′′						144.40		139.22
6′′′′						109.15		108.99
7′′′′						167.22		167.10

#### Jatrophenin-2 (**2**)

Creamy-white amorphous powder (27 mg). *R*_f_ = 0.21 (S1), 0.57 (S2) on PC; deep purple spot turned to pink-red, indigo-red, and blue color with KIO_3_, HNO_2_, and FeCl_3_ spray reagents, respectively. UV (MeOH): *λ*_max_ 216,260, 285sh; (+ MeOH + NaOMe): 210, 240sh,325 nm. ^1^H and ^13^C NMR data (500/125 MHz, DMSO-*d*_6_) were presented in Table 1, Fig. S3 and Fig. S4.

### Antibacterial activity of 1 and 2 and their impact on the in vitro normal cell line model

#### Antibacterial activity and MIC against BKP-122

Results, in Table 2, revealed potent antibacterial activity for the extracted compounds (**1** &** 2**) against the BKP-122 isolate. The assessment of the inhibitory zones showed that the effectiveness of **1** and **2** was slightly equal. The MIC of compounds 1and 2 was recorded at 2.39 µg/ml and 1.87 µg/ml, respectively. In comparison with the standard antimicrobial drugs, Ceftazidime, Gentamicin, and Ertapenem showed a resistant pattern to the BKP-122, as shown in Table 2, and previously evidenced^17^

**Table 2:** Inhibition zones and MIC values of compounds **1** and** 2** against the BKP-122 isolate.

Sample	Inhibition zone diameter (mm)	MIC (µg/ml) Mean ± SD
1	20	2.39 ± 0.875
2	18	1.87 ± 0.625
Ceftazidime	13	16.00 ± 1.30
Gentamicin	11	16.00 ± 1.80
Ertapenem	14	4.00 ± 1.60

MIC: Minimum inhibitory concentration. SD: Standard Deviation.

The statistical analysis showed no significant difference between the MIC means of both compounds **1** and **2** (*p*-value = 0.8137).

#### Cytotoxic effect against normal cells

The obtained MICs of both compounds were promising to continue the investigations. Both tannins were evaluated to check their impact on normal cell viability, where Table 3 is displaying their results. The examination was applied at a concentration range starting with more than 50-fold (125 µg/ml) the obtained MIC against BKP-122*.* Fortunately, neither compound did not exert any toxicity against the normal model cells 48 h post-treatment and up to 125 µg/ml. On the other hand, compound **1** showed only 5% toxicity after 72 h, while **2** was not toxic up to the same concentration mentioned above.

**Table 3:** Cytotoxic effect of compounds **1** and **2** against normal cells.

Sample (Concentration µg/ml)	Toxicity, %	
	after 48 h	after 72 h
1 (125)	0.0	5.0 ± 0.0.6
2 (125)	0.0	0.0
Dox. (2.5)	73.5 ± 1.34	N
Dox. (1.2)	N	75.6 ± 0.82

Dox.: Doxorubicin; N: Not applied

#### Antibiofilm activity against BKP-122

**Fig. 1:**
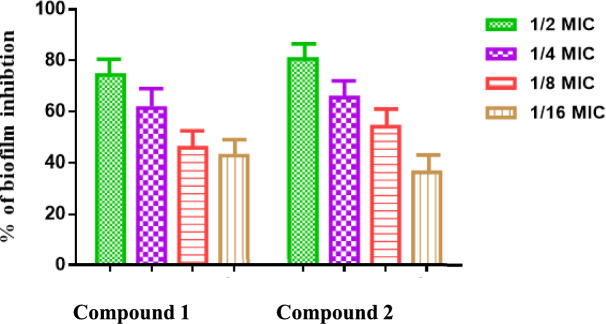
Inhibition of biofilm formation for BKP-122 by **1** and **2** at different sub-MICs (µg/ml).

The microtiter plate assay revealed significant antibiofilm activity for compounds **1** and **2**. At 1/2 MIC, biofilm inhibition reached 74% and 81% respectively. This effect diminished with decreasing concentrations, reaching a minimum of 42% and 36% inhibition at 1/16 MIC. These results demonstrate a clear dose-dependent reduction in biofilm formation, as illustrated in Fig. 1.

#### Effect on the transcriptome genes involved in the biofilm formation of BKP-122

**Fig. 2:**
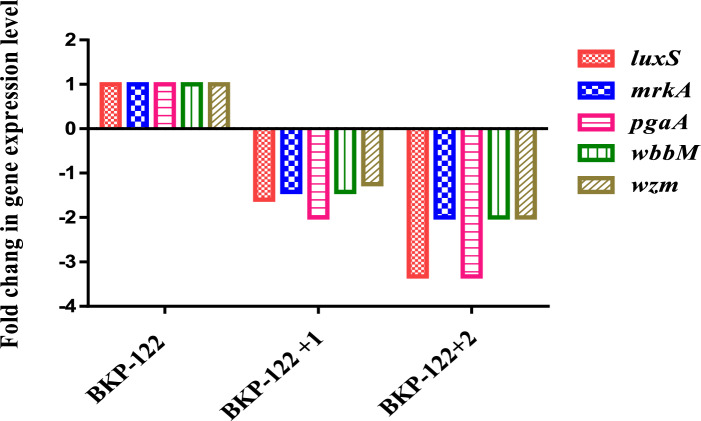
Relative gene expression levels after treatment with 1/2 the MICs (µg/ml) of compounds **1** and **2**. The fold change equaled (2^−ΔΔCT^) as indicated in material and methods section. BKP-122: biofilm-forming *K. pneumoniae* clinical isolate; MIC: Minimum inhibitory concentration.

By comparing the expression profile of genes governing biofilm formation using RT-PCR, the effect of **1** and **2** at ½ -MIC against BKP-122 was evaluated with the untreated sample (Positive control). According to the current study, compound **1** downregulated the expression of genes linked to biofilms *luxS, mrkA, pgaA, wbbM,* and *wzm* genes by 1.60, 1.42, 2.00, 1.42, and 1.25 folds, respectively, as compared to the untreated sample (Positive control). However, compound **2** downregulated the expression of genes linked to biofilms as *luxS, mrkA, pgaA, wbbM,* and *wzm*, by 3.33, 2.00, 3.33, 2.00, and 2.00 folds, respectively, as compared to the untreated sample (Positive control) (Fig. 2).

### Molecular docking study

**Fig. 3:**
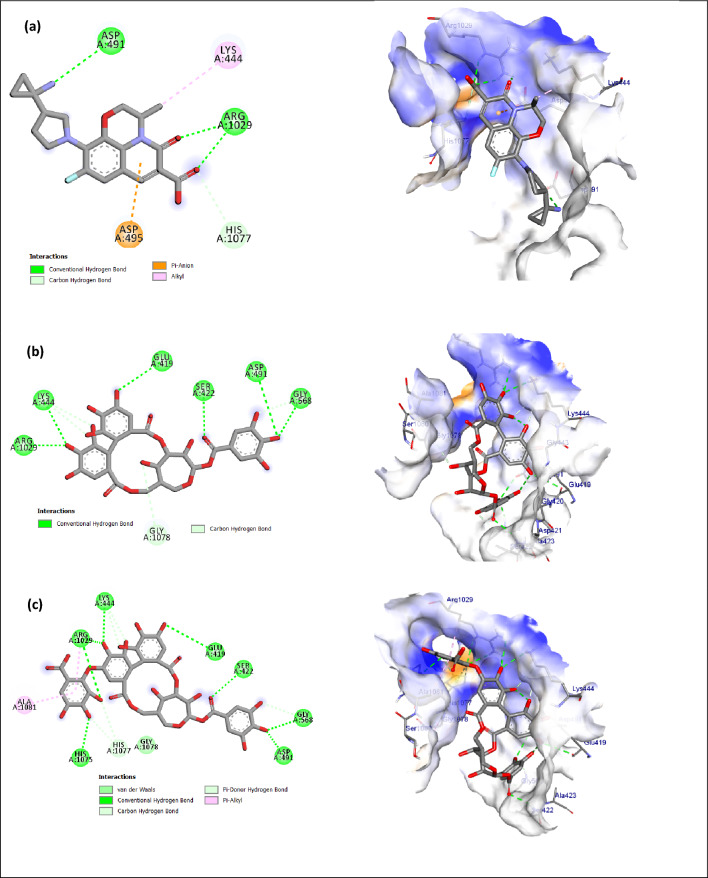
2D and 3D illustrations of the expected binding modes for (**a**) co-crystal ligand (Y21), (**b**): compound **1**, (**c**): compound **2** with Topoisomerase IV active site.

**Fig. 4:**
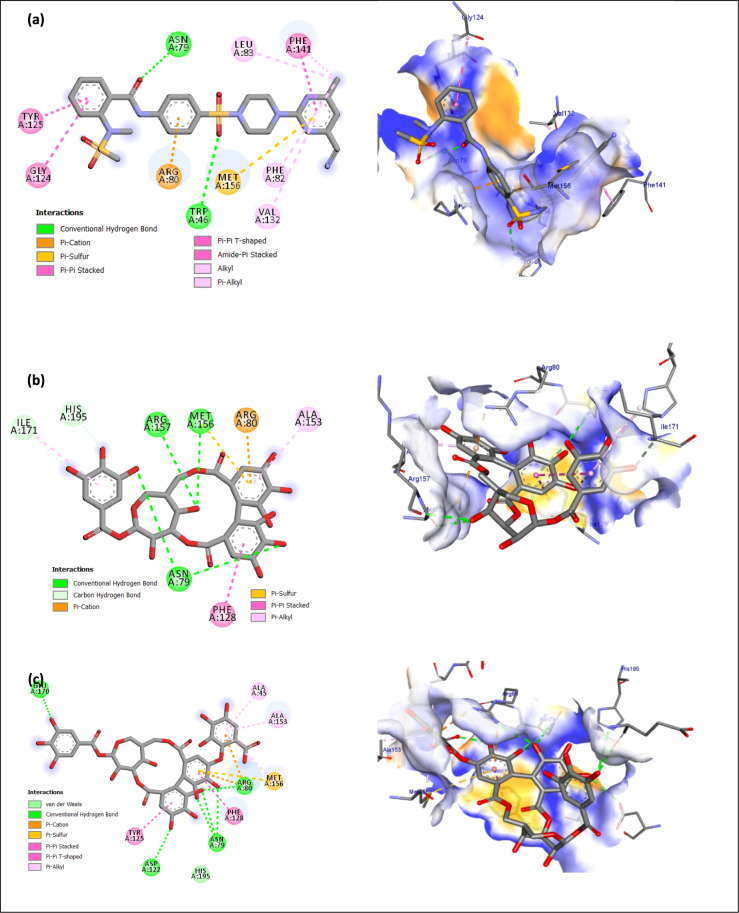
2D and 3D illustrations of the expected binding modes for (**a**) co-crystal ligand (VTF), (**b**): compound **1**, (**c**): compound **2** with KPLpxH active site.

**Fig. 5:**
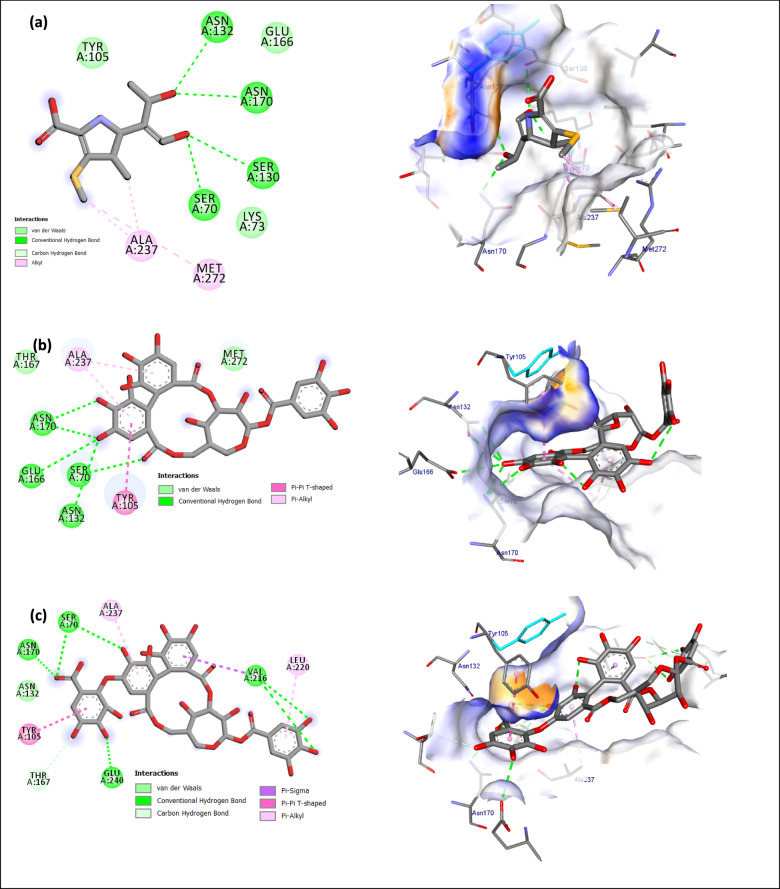
2D and 3D illustrations of the expected binding modes for (**a**) co-crystal ligand (MER), (**b**): compound **1**, (**c**): compound **2** with β-lactamase active site.

Compounds **1** and **2** were precisely situated inside the active sites of the Topoisomerase IV, KPLpxH, and *β*-lactamase enzymes, as demonstrated in Table 4 and Figures 3, 4 and 5, Table S2. The binding affinities of compounds **1** and **2** for Topoisomerase IV were assessed as −8.3 and −9.7 kcal/mol, respectively, in contrast to the co-crystal ligand (**Y21**), which exhibited a binding affinity of −6.7 kcal/mol. compounds **1** and **2** were securely situated in the active site of KPLpxH, exhibiting docking energies of −8.2 and −9.7 kcal/mol, respectively. Additionally, the docked **1** and **2** demonstrated reduced binding energies to β-lactamase (−10 and −9.7 kcal/mol) in contrast to the co-crystal ligand (MER) (−5.5 kcal/mol). The co-crystallized ligands were re-docked into their respective enzymes, resulting in root-mean-square deviations (RMSD) of 0.15 Å for Topoisomerase IV, 0.45 Å for KPLpxH, and 0.53 Å for β-lactamase, when comparing the docked and co-crystallized ligands (Figs. S5–S7, Table S2). This confirms the effectiveness of the utilized docking mechanism.

**Table 4:** Interaction energy calculated using docking calculations of co-crystal ligands, **1** and **2,** with Topoisomerase IV, KPLpxH, and *β*-lactamase enzymes.

Enzymes	Energy score (kcal/mol)		
	Co-crystal ligand	1	2
Topoisomerase IV	–6.7	–8.3	–9.7
KPLpxH	–9.5	–8.2	–9.7
*β*-lactamase	–5.5	–10	–9.4

## Discussion

**Fig. 6:**
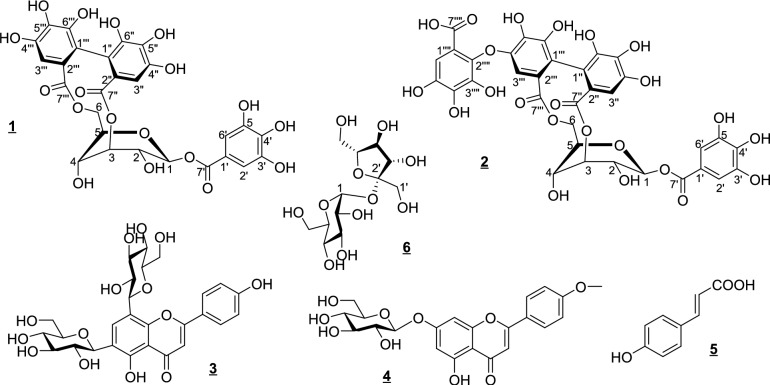
Structural formulas of isolated compounds (**1**–**6**).

Depending on a main polyamide column followed by many separation steps on Sephadex LH-20 columns for the promising collective fractions, 6 metabolites were isolated from the MSP of the total aqueous 70% MeOH-extract from *J. integerrima* Jacq. flowers. The structural formulas were established according to their chromatographic properties and comparison of the spectroscopic data with previous literature of structurally related compounds as 1-*O*-galloyl-3,6-hexahydroxydiphenoyl-**D**-B_1,4_-glucopyranose (Jatrophenin-1, **1**), 1-*O*-galloyl-3,6-valoneoyl-**D**-B_1,4_-glucopyranose (Jatrophenin-2, **2**), vicenin-2 (**3**), acacetin7-*O*-*β*-**D**-glucopyranoside (**4**), (*E*)-*p*-coumaric acid (**5**), and sucrose (**6**)^35–39^, Fig. 6.

Compounds **1** and **2** disclosed almost the same chromatographic characters (*R*_f_-values, fluorescence under UV light, and responses toward specific spray reagents^40^, and UV-spectral data^41^ of two galloyl-HHDP-ellagitannins. In the aromatic region, each of the two ^1^H NMR spectra (Figs. S1, S3) showed a typical 2H–singlet at *δ*_H_ 7.01 ppm for H-2′/6′ of a galloyl ester. Alongside, the presence of one (*R*)-HHDP moiety in the structure of **1** was deduced from its two distinct 1H–singlets at *δ*_H_ 6.56 ppm (H-3′′′) and 6.49 ppm (H-3′′). However, the observation of three singlets at *δ*_H_ 6.92 (H-3′′′′), 6.56 (H-3′′), and 6.49 (H-3′′′) were assignable for one (*R*)-valoneoyl moiety in the structure of **2**^41,42^. Such documents for the phenoyl esters were further supported by the characteristic 5, 14 and 21 ^13^C-signals of each galloyl, HHDP, and valoneoyl ester, respectively, including the carboxyl-carbonyl signals at about *δ*_C_ 165.3 ppm (galloyl), 167.58, and 167.22 ppm (HHDP), and 167.96, 167.58, and 167.22 ppm (valoneoyl).

Because of its known configurational and conformational flexibility in the molecules of tannins, the glucose core was proved to be a *β*-B_1,4_-glucopyranose conformer in both **1** and **2** and not *β*-^4^C_1_- or *β*-^1^C_4_-pyranose due to the *δ-*values, *J-*extents of vicinal ^1^H-^1^H-couplings, and corresponding splitting systems in ^1^H and ^13^C NMR spectra (Figs. S1–S4)^35,39,43,44^. The intrinsic *J*_1,2_ values (7, 7.5 Hz) were another evidence confirming the *β*-B_1,4_-pyranose structure, not ^1^C_4_ of *J*_1,2_ in the range 2–3.5 Hz^43^. That was also agreed with the recording H-2 as br d comprising large *J*_*2*ax-1ax_ (> 7 Hz) and small *J*_2ax-3 eq_ (not resolved). The equatorial configuration for H-3, H-4, and H-5 was deduced from their splitting forms to give further documentation of a *β*-B_1,4_-conformer for the glucose unit in both **1** and **2** structures. Their galloylation on OH-1 was evidenced from the typical downfield shift of the H-1-doublet at about *δ*_H_ 6.20 ppm^35^. Moreover, the bi-functional acylation of OH-3 and OH-6 on glucose core with HHDP or valoneoyl was followed from a similar downfield shift for H-3 (br s), H-6a (dd), and H-6b (dd) to *δ*_H_ 4.59, 4.23, and 3.96 ppm, respectively. The characteristic *α*-downfield shift (Δ ~  + 3 ppm) of C-1 (92.65), C-3 (≈77.91), and C-6 (64.60) and *β*-upfield shift of C-2 (≈72.00) and C-4 (62.60) were proved finally the acylation of OH-1, OH-3, and OH-6 pattern in both compounds^39,43^. Also, it was recommended that the HHDP and valoneoyl moieties be attached, in the same mode, to OH-3 and OH-6 due to the high consistency of the *δ*_H_ and *δ*_C_ values for the corresponding glucose core resonances in both **1** and **2**. It was worth mentioning that the (*S)*-configuration of the phenoyl residues (HHDP, Valoneoyl) must be revised or modified into (*R)*-type because of the characteristic downfield and very close *δ*_H_ values of H-3″ and 3′″ (6.49 and 6.56 ppm, Δ 0.07 ppm), where in case (*S)*-configuration the reported difference is commonly Δ ≥ 0.2 ppm with a relative upfield location. The full interpretation of all ^1^H and ^13^C resonances of **1** and **2** was achieved by a careful matching with corresponding data reported for structurally related compounds^39,43,44^ and their own data, has been confirmed by 2D NMR, isolated once before in nature as new tannins from *Euphorbia cotinifolia* L. by Marzouk et al., 2012. Thus, **1** and **2** were established as 1-*O*-galloyl-3,6-(*R*)-hexahydroxydiphenoyl-**D**-B_1,4_-glucopyranose (Jatrophenin-1) and 1-*O*-galloyl-3,6-(*R*)-valoneoyl-**D**-B_1,4_-glucopyranose (Jatrophenin-2).

In the face of rising antibiotic resistance, particularly among Gram-negative bacteria such as *Escherichia coli* and *Klebsiella pneumoniae*, there is an urgent need for innovative antimicrobial strategies^25,45–47^. Emerging approaches in antibiotic development include the use of probiotics, bacteriophages, anti-quorum-sensing agents, and plant-derived compounds^23,46,47^. Pentagalloylglucose, a well-known hydrolysable tannin, has been reported to possess a variety of biological activities, including antimicrobial, antiviral, anticancer, antioxidant, and antidiabetic effects^48^. In this study, two promising tannins were isolated and their in vitro antibacterial activity was thoroughly evaluated against the multidrug-resistant strain BKP-122. Antibacterial efficacy was determined by measuring the diameters of the inhibition zones. The results demonstrated strong antibacterial effects of compounds 1 and 2 against BKP-122, especially when compared to standard antibiotics such as ceftazidime, gentamicin, and ertapenem, which showed resistance according to CLSI guidelines (2025). These findings are consistent with previous studies showing that tannins typically exert bacteriostatic effects on both Gram-positive and Gram-negative bacteria, although the latter tend to be less susceptible^49^.

The minimum inhibitory concentrations (MICs) for compounds 1 and 2 were found to be 2.39 µg/mL and 1.87 µg/mL, respectively. These results align with earlier research highlighting the antimicrobial effects of *Jatropha* species against various bacterial strains, including both Gram-negative^50^ and Gram-positive bacteria^51^. Notably, extracts from *J. curcas* have previously demonstrated stronger antibacterial activity against BKP-122 than gentamicin^52,53^.

The studied compounds are structurally related to gallotannins, a class of hydrolysable tannins known for their antimicrobial properties^54,55^. Besides, corilagen, which is a more closely related conformer, differs only in the ^1^C_4_-glucose core, confirming in *vitro* and in *vivo* antibacterial activity against *S. pneumoniae*^56^. Moreover, comparable antimicrobial properties were reported by Masota et al.^49^**,** who found that the antibacterial efficacy of penta-galloylglucoses, as measured by the MIC or the minimum regrowth concentration (MRC), ranges from 2 to 64 µg/ml, exhibiting activity against various reference and multidrug-resistant (MDR) strains of *E. coli* and *K. pneumoniae*. Due to the substantial structural dissimilarity between galloylglucoses and conventional antibiotic substrates like β-lactams, it is improbable that the differences in how MDR bacteria respond to galloylglucoses are a result of direct enzyme–substrate interactions. Therefore, the variability in bacterial susceptibility may instead be influenced by other factors related to resistance enzymes, as well as the relative abundance of aromatic amino acids^49^.

The present study investigated the effects of ellagitannins 1 and 2 at sub-MIC concentrations on the development of BKP-122 biofilms. Notably, biofilm formation was reduced in a dose-dependent fashion. At half the MIC, compounds 1 and 2 inhibited biofilm formation by 74% and 81%, respectively. These findings align with those of Gamal El-Din et al.^50^, who demonstrated the antibiofilm properties of *Jatropha* species (*J. integerrima*, *J. gossypifolia*, and *J. roseae*) against the Gram-negative bacterium *E. coli*. Additionally, galloylglucoses have been reported to inhibit biofilm formation, bacterial adhesion, the expression of surface transport proteins, toxins, and extracellular enzymes^57^.

The present study provides deeper mechanistic insights into how the identified rare tannins affect five biofilm-associated genes in the BKP-122 strain, contributing to a reduction in its virulence. Compound 1 was found to downregulate the expression levels of the genes *luxS*, *mrkA*, *pgaA*, *wbbM*, and *wzm* by 1.60, 1.42, 2.00, 1.42, and 1.25 fold, respectively, compared to the untreated bacterial control. In contrast, compound 2 demonstrated even greater efficacy, significantly reducing the expression of the same genes by 3.33, 3.33, 2.00, and 2.00 fold, respectively, indicating a stronger inhibitory effect on gene expression relative to compound 1.

Eradicating the formed biofilm requires continuous high doses of the used antibiotic. Nevertheless, this strategy sometimes does not succeed in overcoming the biofilm infections^58^. Conventional medication against such infections is particularly challenging since the doses that are sub-lethal to the biofilm must be administered safely to the patient. Moreover, the reported literature suggested using combinations of antibiotics, rather than single therapy, to eliminate the formed biofilms^59–64^. The present results introduced potential molecules in the concerned era. Both of the isolated tannins possess the power to fight such types of pathogens at a safe concentration range.

Topoisomerase IV (TopoIV) is an essential bacterial enzyme that resolves newly duplicated DNA and facilitates the division of daughter chromosomes. In bacteria, DNA replication and division occur simultaneously. TopoIV must consistently eliminate inter-DNA connections throughout replication^65^. Compounds **1** and **2**, along with the co-crystal ligand (**Y21**), are strategically located inside the active binding site of the BKP-122 topoisomerase IV enzyme (Fig. 3). Figure 3a illustrates the two-dimensional and three-dimensional models of the docked complex of compound **Y21**, highlighting the residues Arg1029, Asp491, and His1077, which participate in five hydrogen bonds, alongside the Lys44 residue, which exhibits hydrophobic interaction (Table S2). The docking score for **Y21** is –6.7 kcal/mol, in contrast to **1** and **2**, which have scores of –8.3 and –9.7 kcal/mol, respectively (Fig. 5). Compound **1** was accurately positioned in the binding site of TopoIV, forming 11 hydrogen bonds with critical amino acids: Ser422 (2.52 Å), Lys444 (2.75, 3.48, and 3.61 Å), Gly568 (2.37, 3.47 Å), Arg1029 (3.02, 2.38 Å), Glu419 (3.37 Å), Asp491 (3.35 Å), and Gly1078 (3.66 Å) (Fig. 3b, Table S2). Moreover, **2** exhibited the highest binding affinity, establishes 16 hydrogen bonds with Ser422 (2.44 Å), Lys444 (2.83, 3.49, and 3.59 Å), Gly568 (2.47, 3.49 Å), Arg1026 (2.77 Å), Arg1029 (3.08, 2.65, and 2.38 Å), His1075 (2.73 Å), Glu419 (3.34 Å), Asp491 (3.39 Å), and His1077 (3.39, 4.05, 4.14 Å). Additionally, it engages in 2 Pi-alkyl interactions with Arg1029 and Ala1081 (Fig. 3c, Table S2), which constitute a substantial driving force for complex formation.

The outer membrane of Gram-negative pathogens is characterized by the presence of lipopolysaccharide (LPS) or lipo-oligosaccharide (LOS) protruding from its outer monolayer. Lipid A serves as the hydrophobic membrane anchor of LPS/LOS and constitutes the active component of the bacterial endotoxin responsible for Gram-negative septic shock during bacterial infection. The Raetz pathway is essential for the biosynthesis necessary for bacterial viability and fitness within the human host. LpxH is a crucial, late-stage lipid A enzyme that catalyzes the hydrolysis of the pyrophosphate group from UDP-2,3-diacylglucosamine (UDP-DAGn), resulting in the formation of 2,3-diacylglucosamine 1-phosphate (lipid X) and UM^66^. The co-crystal ligand (VET) exhibited two hydrogen bonds with Trp46 (3.36 Å) and Asn79 (3.37 Å), and also established eight hydrophobic contacts with Arg80, Met156, Phe141, Tyr125, Gly124, Leu83, Phe80, and Phe141 (Fig. 4a, Table S2). Compound **1** engaged with critical residues through six hydrogen bonds: Asn79 at distances of 3.31 and 3.37 Å, Met156 at a distance of 2.95 Å, Arg157 at a distance of 3.30 Å, Ile171 at a distance of 3.31 Å, and His195 at a distance of 3.54 Å. Furthermore, **1** exhibited five hydrophobic bonds (Fig. 4b and Table S2). Compound **2** exhibited the highest docking score of –9.7 kcal/mol, in contrast to **1** and VET, which scored –8.2 kcal/mol and –9.5 kcal/mol, respectively (Table 4). Compound** 2** formed seven hydrogen connections with Asn79 (3.21, 3.31, 3.39 Å), Arg80 (3.30 Å), Glu170 (3.37, 2.97 Å), and Asp122 (3.28 Å). Furthermore, **2** exhibited two Pi-cation bonds, one Pi-sulfur bond, two Pi-Pi stacking links, and three Pi-alkyl bonds (Fig. 4c and Table S2).

The increasing prevalence of antibiotic resistance in *K. pneumoniae* isolates has emerged as a major global issue. *β*-lactamases, the enzymes that confer resistance to *β*-lactam antibiotics, are classified into classes A, B, C, and D according to the Ambler categorization system. *β*-lactamases are the principal agents of bacterial resistance to penicillins and cephalosporins^67^. In class A *β*-lactamase, the *β*-lactam binding site, which includes the catalytic Ser70, is situated between two domains: one is α-helical, while the other features an α/β motif with a five-stranded antiparallel *β-*sheet^68^. The co-crystal ligand (MER) is optimally situated within the active site of *β-*lactamase, demonstrating a docking score inferior to that of **1** and **2**. MER interacts within the binding site via five hydrogen bonds (Ser70, Asn170, Ser130, and Asn132) and alkyl interactions (Ala237, Met272) (Fig. 5a, Table S2). Compound **1** demonstrated six hydrogen bonds with critical amino acids (Ser70, Asn132, Asn170, and Glu166) and three Pi bonds (Tyr105 and Ala237) (Fig. 5b, Table S2). Additionally, compound **2** exhibited nine hydrogen bonds with Ser70, Asn170, Glu240, Val216, and Thr167, along with four Pi bonds involving Val216, Tyr105, Ala237, and Leu220 (Fig. 5c, Table S2).

## Conclusion

In conclusion, this study offers strong molecular evidence that *Jatropha integerrima*'s isolated ellagitannins are potent antimicrobial and antibiofilm agents in addition to validating the plant’s traditional medicinal use. Jatrophenin-1 and Jatrophenin-2 provide an attractive and practical therapeutic pathway to fight antibiotic resistance and biofilm-related infections by directly interfering with the genetic machinery regulating virulence and resistance in MDR pathogens like *K. pneumoniae.* Furthermore, the docking studies revealed a robust affinity for the Topoisomerase IV, KPLpxH, and β-lactamase binding site. In vivo studies may be beneficial for application of their use to treat several infections by demonstrating their antibacterial and antibiofilm activities in in-vivo models against several resistant bacteria.

## Supplementary Information

Supplementary Information.

## Data Availability

Data is provided within the manuscript or supplementary information files.
