# Supplementary material for: The gene-modulating power of Tannins isolated from Jatropha integerrima flowers on the transcriptomic profile of multidrug-resistant Klebsiella pneumoniae
Source: Sci Rep. 2026 Jan 14;16:2074. doi: 10.1038/s41598-025-32490-0 (PMC12808767; doi:10.1038/s41598-025-32490-0)
Supplement: Supplementary file 1 — Supplementary Information. [file 41598_2025_32490_MOESM1_ESM.docx]

**The Gene-Modulating Power of Tannins Isolated from *Jatropha Integerrima* Flowers on the Transcriptomic Profile of Multidrug-Resistant *Klebsiella Pneumonia***

Fatma S. Mahrous^1^, Shimaa M. Khalifa^1, *^, Fatma Sayed Abdel-Aal Farag^1^, Mona Shaban E. M. Badawy^2^, Omnia Karem M. Riad^2^, Mona H. Ibrahim^3^, Maha M. Soltan^4^, Mohamed Marzouk^5, *^

***^1^****Department of Pharmacognosy and Medicinal Plants, Faculty of Pharmacy (Girls), Al-Azhar University, Cairo 11754, Egypt*

*^2^Department of Microbiology and Immunology, Faculty of Pharmacy (Girls), Al-Azhar University, Cairo 11754, Egypt*

*^3^Department of Pharmaceutical Medicinal Chemistry and Drug Design, Faculty of Pharmacy (Girls), Al-Azhar University, Cairo 11754, Egypt*

*^4^Biology Unit, Central Laboratory for Pharmaceutical and Drug Industries Research Institute, Chemistry of Medicinal Plants Department, National Research Centre, 33 El Bohouth St. (Former El-Tahrir St.), Dokki, Cairo 12622, Egypt;* [mahasoltan@netscape.net](mailto:mahasoltan@netscape.net)

*^5^Chemistry of Tanning Materials and Leather Technology Department, Chemical Industries Research Institute, National Research Centre, 33 El-Bohouth St. (Former El-Tahrir St.), Dokki, Cairo 12622*

***Correspondence:**

**Mohamed Marzouk; E-mail:** [**msmarzouk@yahoo.co.uk**](mailto:msmarzouk@yahoo.co.uk)**, ORCID No.: 0000-0002-1344-0617**

**Shimaa M. Khalifa; E-mail:** [**ShimaaKhalifa.52@azhar.edu.eg**](mailto:ShimaaKhalifa.52@azhar.edu.eg)**, ORCID No.: 0000-0003-3381-7036**

**Supplementary Materials**

**Appendix**

| Item | Subject |
| --- | --- |
| Table S1 | Primer sequences for evaluation of the expression levels of biofilm formation genes |
| Table S2 | Docking binding free energy of the compounds **1** and **2** and the co-crystallized ligands against Topoisomerase IV., KPLpxH and β-lactamase enzymes |
| Figure S1 | ^1^H NMR spectrum of tannin **1** in DMSO-*d_6_* |
| Figure S2 | ^13^C NMR spectrum of tannin **1** in DMSO-*d_6_*. |
| Figure S3 | ^1^H NMR spectrum of tannin **2** in DMSO-*d_6_*. |
| Figure S4 | ^13^C NMR spectrum of tannin **2** in DMSO-*d_6_*. |
| Figure S4 continue | Expansions of the ^13^C NMR spectrum  of tannin **2** in DMSO-*d_6_* (aromatic and aliphatic regions). |
| Figure S5 | The root mean square deviation between the original and docked poses of the co-crystal ligand (Y21) of Topoisomerase IV(PDB: 7LHZ) was 0.15 Å. |
| Figure S6 | The root mean square deviation between the original and docked poses of the co-crystal ligands (VTF) of KPLpxH(PDB: 8QK2) was 0.45 Å. |
| Figure S7 | The root mean square deviation between the original and docked poses of the co-crystal ligands (MER) of *β*-lactamase (PDB: 2ZD8) was 0.53 Å. |

**Table S1**. Primer sequences for evaluation of the expression levels of biofilm formation genes.

| **Gene** | **Primer sequence** | **Tm (°C)** | **References** |
| --- | --- | --- | --- |
| *luxS*-F | 5ʹ-AGT GAT GCC GGA ACG CGG-3ʹ | 60 | [[1](#_ENREF_1)] |
| *luxS*-R | 5ʹ-CGG TGT ACC AAT CAG GCT C-3ʹ | 60 |  |
| *mrkA*-F | 5ʹ-ACG TCT CTA ACT GCC AGG C-3ʹ | 60 |  |
| *mrkA*-R | 5ʹ-TAG CCC TGT TGT TTG CTG GT-3ʹ | 60 | [[2](#_ENREF_2)] |
| *pgaA*-F | 5ʹ-GCA GAC GCT CTC CTA TGT C-3ʹ | 60 | [[1](#_ENREF_1)] |
| *pgaA*-R | 5ʹ-GCC GAG AGC AGG GGA ATC-3ʹ | 60 |  |
| *wbbM*-F | 5ʹ-ATG CGG GTG AGA ACA AAC CA-3ʹ | 60 |  |
| *wbbM*-R | 5ʹ-AGC CGC TAA CGA CAT CTG AC-3ʹ | 62 |  |
| *wzm*-F | 5ʹ-TGC CAG TTC GGC CAC TAA C-3ʹ | 62 |  |
| *wzm*-R | 5ʹ-GAC AAC AAT AAC CGG GAT GG-3ʹ | 62 |  |
| *23S rRNA*-F | 5ʹ-ATC GTA CCC CAA ACC GAC AC-3ʹ | 62 |  |
| *23S rRNA*-R | 5ʹ-TTC TCC CGA AGT TAC GGC AC-3ʹ | 62 |  |

T_m_: melting temperature

Table S2. Docking binding free energy of the compounds **1** and **2** and the co-crystallized ligands against Topoisomerase IV., KPLpxH and β-lactamase enzymes

| **Enzyme** | **Bond type /Involved amino acids/ Distance in (Å)** | | |
| --- | --- | --- | --- |
|  | **Co-crystal ligand** | **1** | **2** |
| Topoisomerase | Conventional Hydrogen Bond/Arg1029/2.81  Conventional Hydrogen Bond/Arg1029/2.59  Conventional Hydrogen Bond/Arg1029/2.86  Conventional Hydrogen Bond/Asp491/3.34  Carbon Hydrogen Bond/His1077/3.36  Pi-Anion/Asp495/3.65  Alkyl/Lys444/4.10 | Conventional Hydrogen Bond/Ser422/2.52  Conventional Hydrogen Bond/Lys444/2.75  Conventional Hydrogen Bond/Gly568/2.37  Conventional Hydrogen Bond/Arg1029/3.02  Conventional Hydrogen Bond/Arg1029/2.38  Conventional Hydrogen Bond/Glu419/3.37  Conventional Hydrogen Bond/Asp491/3.35  Carbon Hydrogen Bond/Lys444/3.48  Carbon Hydrogen Bond/Lys444/3.61  Carbon Hydrogen Bond/Gly568/3.47  Carbon Hydrogen Bond/Gly1078/3.66 | Conventional Hydrogen Bond/Ser422/2.44  Conventional Hydrogen Bond/Lys444/2.83  Conventional Hydrogen Bond/Gly568/2.47  Conventional Hydrogen Bond/Arg1026/2.77  Conventional Hydrogen Bond/Arg1029/3.08  Conventional Hydrogen Bond/Arg1029/2.65  Conventional Hydrogen Bond/Arg1029/2.38  Conventional Hydrogen Bond/His1075/2.73  Conventional Hydrogen Bond/Glu419/3.34  Conventional Hydrogen Bond/Asp491/3.39  Carbon Hydrogen Bond/Lys444/3.49  Carbon Hydrogen Bond/Lys444/3.59  Carbon Hydrogen Bond/Gly568/3.49  Carbon Hydrogen Bond/His1077/3.39  Pi-Donor Hydrogen Bond/His1077/4.05  Pi-Donor Hydrogen Bond/His1077/4.14  Pi-Alkyl/Arg1029/5.14  Pi-Alkyl/Ala1081/4.50 |
| KPLpxH | Conventional Hydrogen Bond/Trp46/3.36  Conventional Hydrogen Bond/Asn79/3.37  Pi-Cation/Arg80/3.49  Pi-Sulfur/Met156/5.93  Pi-Pi Stacked/Phe141/3.85  Pi-Pi T-shaped/Tyr125/4.98  Amide-Pi Stacked/Gly124/Tyr125/5.18  Alkyl/Leu83/4.34  Pi-Alkyl/Phe82/5.11  Pi-Alkyl/Phe141/4.79 | Conventional Hydrogen Bond/Asn79/3.31  Conventional Hydrogen Bond/Asn79/3.37  Conventional Hydrogen Bond/Met156/2.95  Conventional Hydrogen Bond/Arg157/3.30  Carbon Hydrogen Bond/Ile171/3.31  Carbon Hydrogen Bond/His195/3.54  Pi-Cation/Arg80/3.61  Pi-Sulfur/Met156/4.16  Pi-Pi Stacked/Phe128/4.14  Pi-Alkyl/Ala153/5.47  Pi-Alkyl/Ile171/5.32 | Conventional Hydrogen Bond/Asn79/3.21  Conventional Hydrogen BondAsn79/3.31  Conventional Hydrogen Bond/Arg80/3.30  Conventional Hydrogen Bond/Glu170/3.37  Conventional Hydrogen Bond/Asn79/3.39  Conventional Hydrogen Bond/Asp122/3.28  Conventional Hydrogen Bond/Glu170/2.97  Pi-Cation/Arg80/3.90  Pi-Cation/Arg80/3.73  Pi-Sulfur/Met156/5.29  Pi-Pi Stacked/Phe128/4.13  Pi-Pi T-shaped/Tyr125/4.96  Pi-Alkyl/Ala45/4.57  Pi-Alkyl/Ala153/4.11  Pi-Alkyl/Met156/5.21 |
| β-lactamase | Conventional Hydrogen Bond/Ser70/3.17  Conventional Hydrogen Bond/Asn170/3.34  Conventional Hydrogen Bond/Ser130/3.17  Conventional Hydrogen Bond/Asn132/3.25  Carbon Hydrogen Bond/Ser130/3.43  Alkyl/Ala237/4.32  Alkyl/Ala237/3.86  Alkyl/Met272/4.97 | Conventional Hydrogen Bond/Ser70/3.36  Conventional Hydrogen Bond/Asn132/3.38  Conventional Hydrogen Bond/Asn170/3.02  Conventional Hydrogen Bond/Asn170/3.19  Conventional Hydrogen Bond/Asn132/3.38  Conventional Hydrogen Bond/Glu166/3.33  Pi-Pi T-shaped/Tyr105/4.60  Pi-Alkyl/Ala237/5.02  Pi-Alkyl/Ala237/4.01 | Conventional Hydrogen Bond/Ser70/3.36  Conventional Hydrogen Bond/Ser70/3.02  Conventional Hydrogen Bond/Ser70/3.32  Conventional Hydrogen Bond/Ser70/3.29  Conventional Hydrogen Bond/Asn170/3.28  Conventional Hydrogen Bond/Glu240/3.28  Conventional Hydrogen Bond/Val216/3.23  Conventional Hydrogen Bond/Val216/3.33  Carbon Hydrogen Bond/Thr167/3.55  Pi-Sigma/Val216/3.90  Pi-Pi T-shaped/Tyr105/4.72  Pi-Alkyl/Ala237/4.52  Pi-Alkyl/Leu220/5.10 |

|  |
| --- |
| Fig. S1. ^1^H NMR spectrum of tannin **1** in DMSO-*d_6_*. |

| 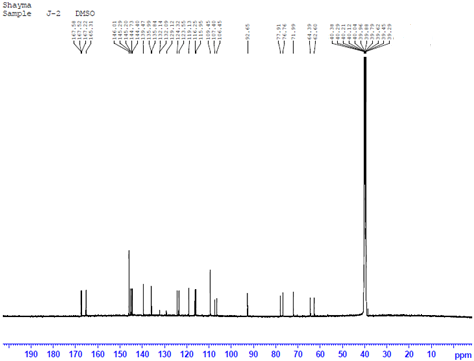 |
| --- |
| Fig. S2. ^13^C NMR spectrum of tannin **1** in DMSO-*d_6_*. |

|  |
| --- |
| Fig. S3. ^1^H NMR spectrum of tannin **2** in DMSO-*d_6_*. |

| 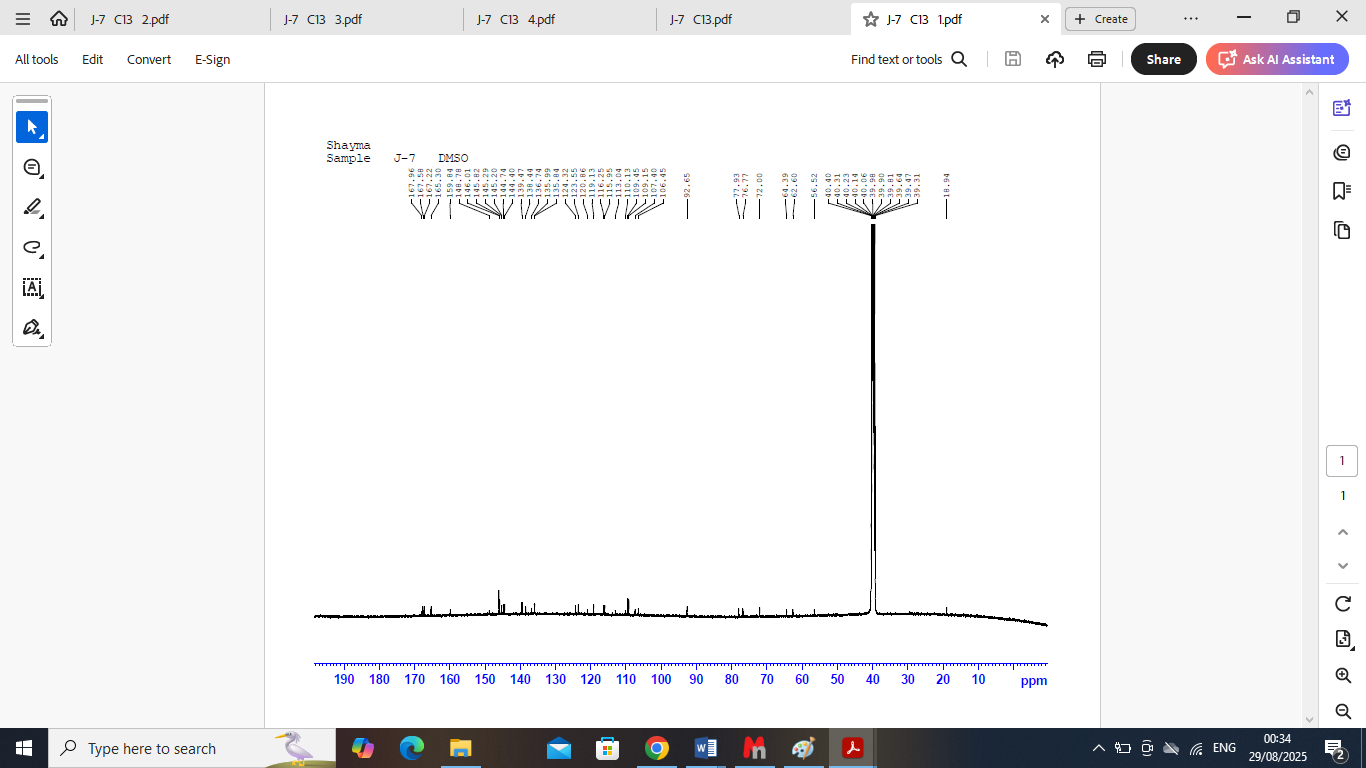 |
| --- |
| Figure S4. ^13^C NMR spectrum of tannin **2** in DMSO-*d_6_*. |

| 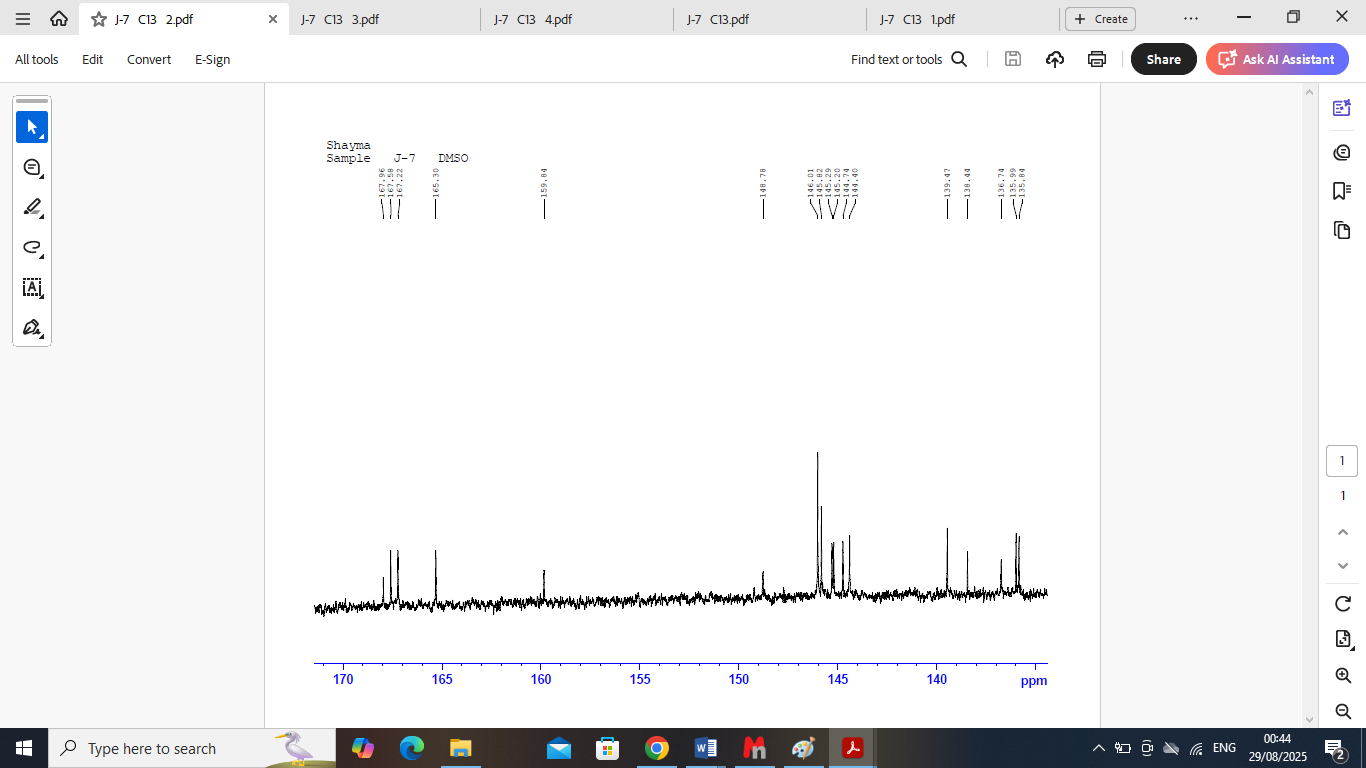 | 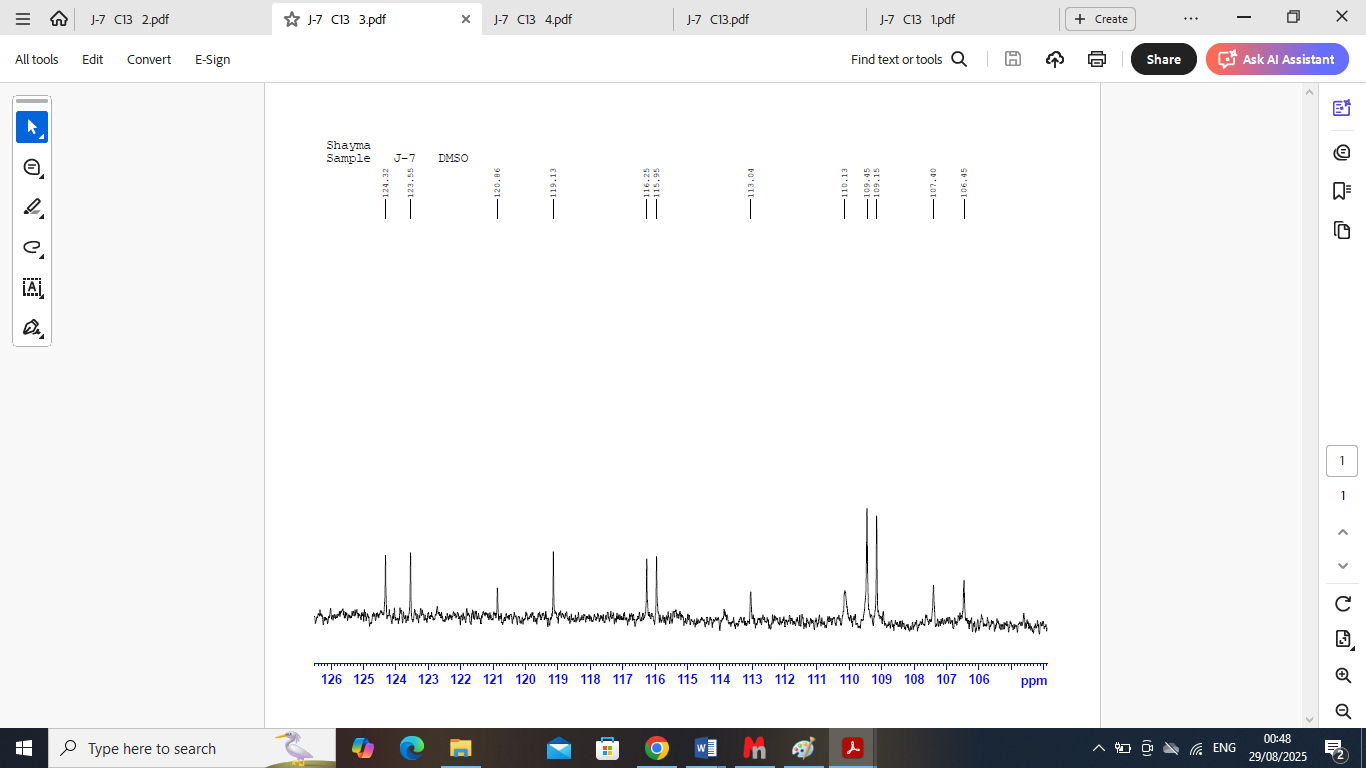 |
| --- | --- |
| 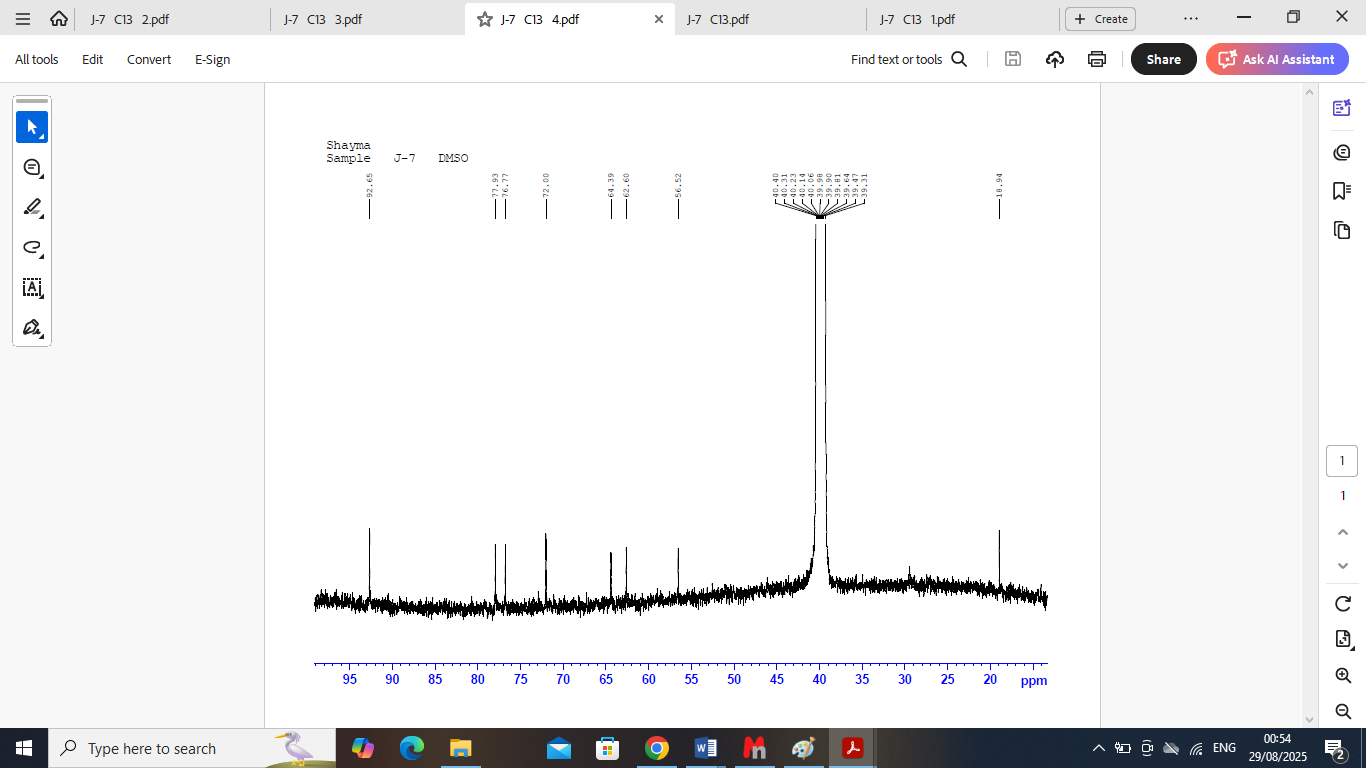 | Fig. S4 continue. Expansions of the ^13^C NMR spectrum  of tannin **2** in DMSO-*d_6_* (aromatic and aliphatic regions). |

| **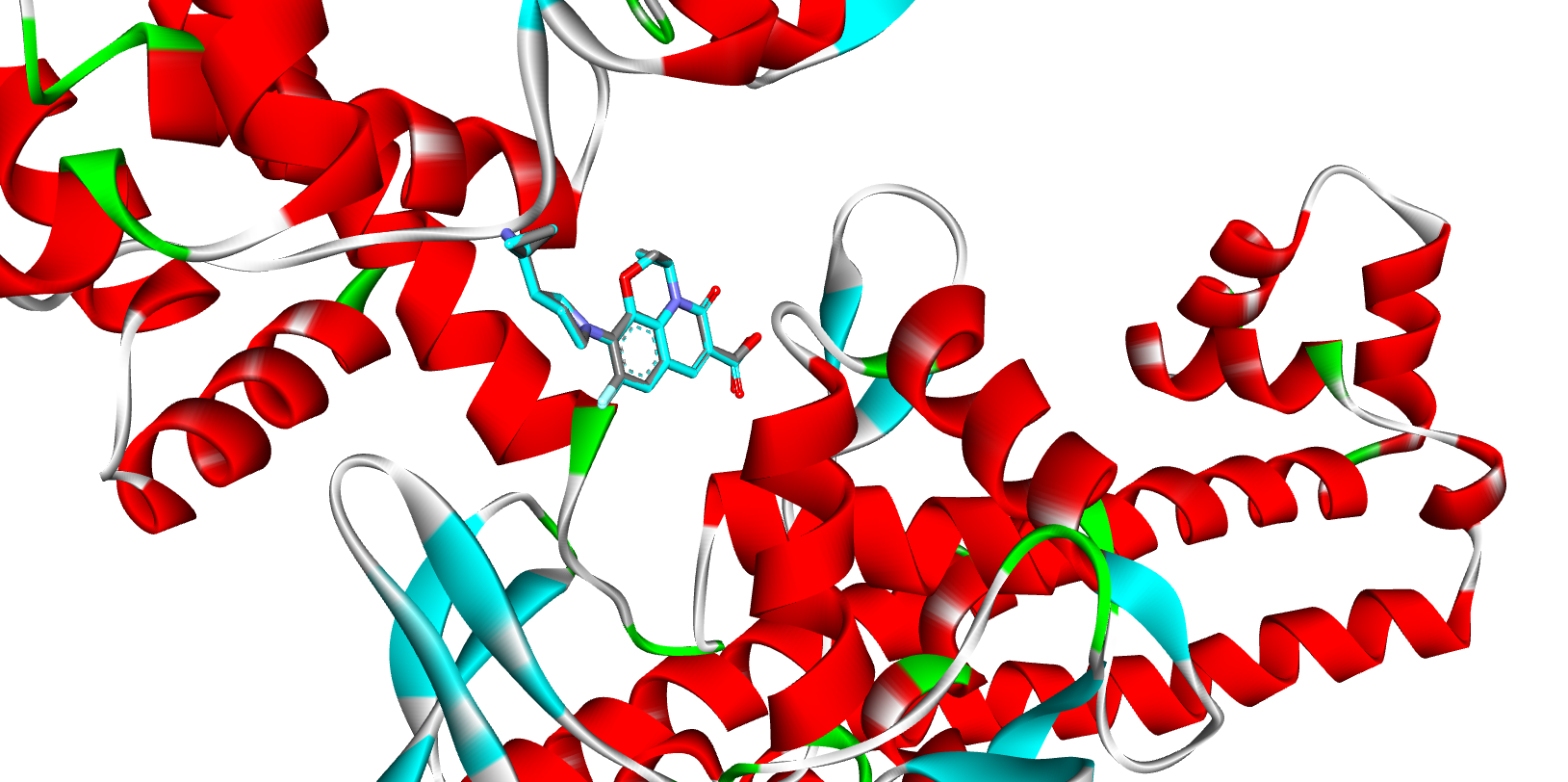** |
| --- |
| Fig. S5. The root mean square deviation between the original and docked poses of the co-crystal ligand (Y21) of Topoisomerase IV(PDB: 7LHZ) was 0.15 Å. |

| 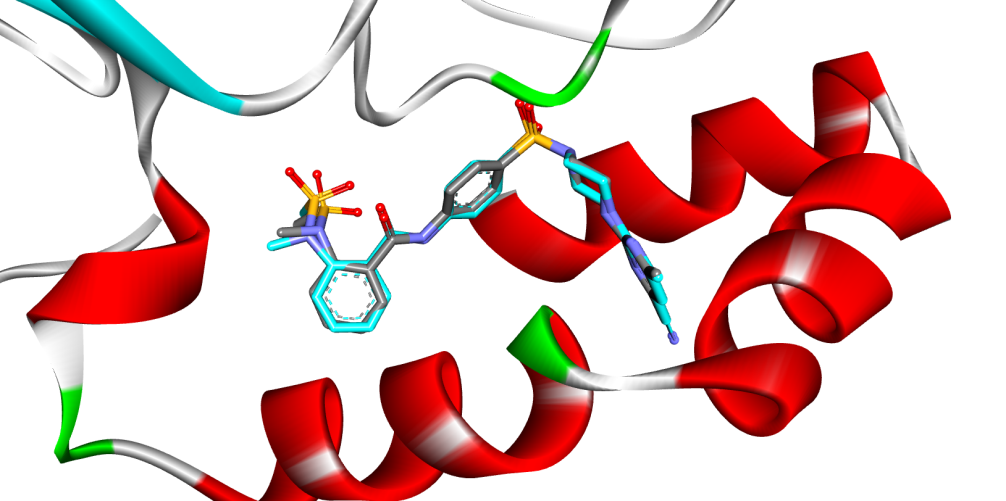 |
| --- |
| Fig. S6. The root mean square deviation between the original and docked poses of the co-crystal ligands (VTF) of KPLpxH(PDB: 8QK2) was 0.45 Å. |

| **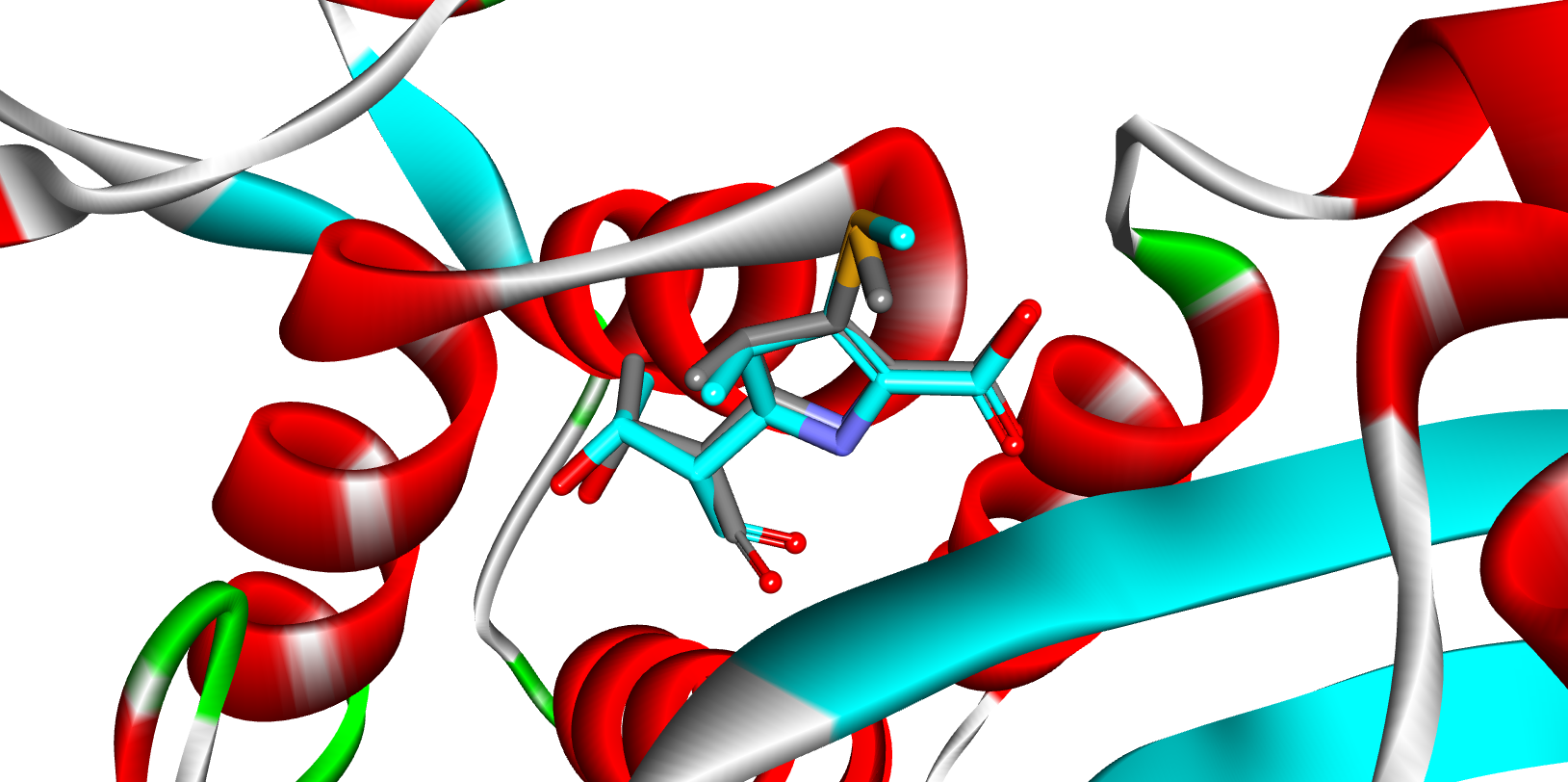** |
| --- |
| Fig. S7. The root mean square deviation between the original and docked poses of the co-crystal ligands (MER) of *β*-lactamase (PDB: 2ZD8) was 0.53 Å. |

1. Vuotto, C., et al., *Biofilm formation and antibiotic resistance in Klebsiella pneumoniae urinary strains.* Journal of applied microbiology, 2017. **123**(4): p. 1003-1018.

2. Wu, C.-C., et al., *Fur-dependent MrkHI regulation of type 3 fimbriae in Klebsiella pneumoniae CG43.* Microbiology, 2012. **158**(4): p. 1045-1056.
